# Functionalized Nanomaterials In Pancreatic Cancer Theranostics And Molecular Imaging

**DOI:** 10.1002/open.202400232

**Published:** 2024-10-21

**Authors:** Yoghalakshmi Nagarajan, Natarajan Chandrasekaran, Venkatachalam Deepa Parvathi

**Affiliations:** ^1^ Department of Biomedical Sciences Faculty of Biomedical Sciences & Technology Sri Ramachandra Institute of Higher Education and Research (SRIHER) Tamil Nadu Chennai 600116 India; ^2^ Senior Professor & Former Director Centre for Nanobiotechnology Vellore Institute of Technology (VIT) Vellore Campus, Tiruvalam road Tamil Nadu Katpadi Vellore 632014

**Keywords:** Targeted therapy, Immunotherapy, Nanomedicine, Theranostics, Functionalized nanoparticles, Pancreatic cancer

## Abstract

Pancreatic cancer (PC) is one of the most fatal malignancies in the world. This lethality persists due to lack of effective and efficient treatment strategies. Pancreatic ductal adenocarcinoma (PDAC) is an aggressive epithelial malignancy which has a high incidence rate and contributes to overall cancer fatalities. As of 2022, pancreatic cancer contributes to about 3 % of all cancers globally. Over the years, research has characterised germline predisposition, the origin cell, precursor lesions, genetic alterations, structural alterations, transcriptional changes, tumour heterogeneity, metastatic progression, and the tumour microenvironment, which has improved the understanding of PDAC carcinogenesis. By using molecular‐based target therapies, these fundamental advancements support primary prevention, screening, early detection, and treatment. The focus of this review is the use of targeted nanoparticles as an alternative to conventional pancreatic cancer treatment due to the various side effects of the latter. The principles of nanoparticle based cancer therapy is efficient targeting of tumour cells via enhanced permeability and retention (EPR) effects and decrease the chemotherapy side effects due to their non‐specificity. To increase the efficiency of existing therapies and modify target nanoparticles, several molecular markers of pancreatic cancer cells have been identified. Thus pancreatic cancer cells can be detected using appropriately functionalized nanoparticles with specific signalling molecules. Once cancer has been identified, these nanoparticles can kill the tumour by inducing hyperthermia, medication delivery, immunotherapy or gene therapy. As potent co‐delivery methods for adjuvants and tumor‐associated antigens; nanoparticles (NPs) have demonstrated significant promise as delivery vehicles in cancer therapy. This ensures the precise internalization of the functionalized nanoparticle and thus also activates the immune system effectively against tumor cells. This review also discusses the immunological factors behind the uptake of functionalized nanoparticles in cancer therapies. Theranostics, which combine imaging and therapeutic chemicals in a single nanocarrier, are the next generation of medicines. Pancreatic cancer treatment may be revolutionised by the development of a tailored nanocarrier with diagnostic, therapeutic, and imaging capabilities. It is extremely difficult to incorporate various therapeutic modalities into a single nanocarrier without compromising the individual functionalities. Surface modification of nanocarriers with antibodies or proteins will enable to attain multifunctionality which increases the efficiency of pancreatic cancer therapy.

## Graphical abstract

Nanoparticles that store and deliver drugs can be used to treat and manage pancreatic cancer. Active targeting by functionalizing nanoparticle with an appropriate molecule is an efficient strategy to boost specificity and affinity. Cancer therapies may be able to target undiscovered pathways by taking advantage of the multifunctional capabilities of functionalized nanoparticles. The control of antitumor immune responses is largely dependent on the tumour microenvironment (TME). The TME is made up of a variety of cells, which encourage tumour cell proliferation, tumour angiogenesis, extracellular matrix remodelling, and general immunosuppression. Targeted therapy aims to lower the immunosuppressive condition in the TME to increase the accuracy and efficacy of the cancer cells response to drug. Functionalized nanoparticles make it possible to deliver anticancer medications precisely to the tumour site, thus minimising any adverse effects from systemic administration.

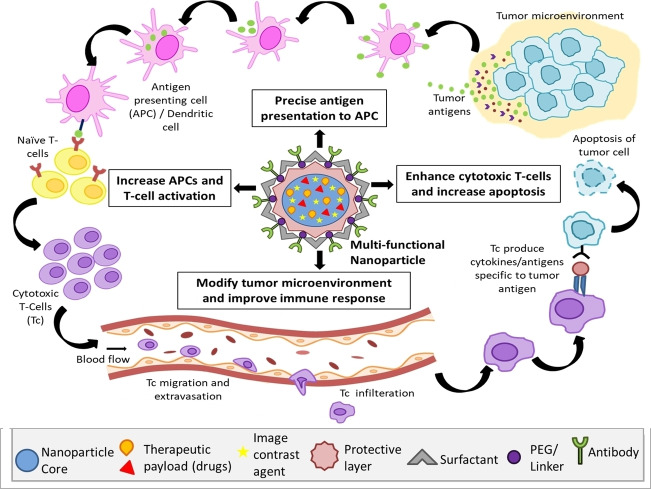

## 1 Introduction

Pancreatic cancer is an aggressive and heterogeneous disease with poor outcome and prognosis. Pancreatic cancer is the 7^th^ most leading cause of cancer death and 14^th^ most common cancer in the world. By 2025, pancreatic cancer is projected to rank third among cancer‐related mortality in Europe, and second by 2030 in the United States.[^1^, ^2^] Poor prognostic factors include lymph‐node metastases, a high tumour grade, a large tumour, high levels of CA 19–9 and persistently elevated postoperative levels of CA 19–9.^[3]^ Pancreatic cancer is one of the dreaded cancers, owing in part to late detection. Surgical resection is usually considered the only possibly curative treatment once the disease has been detected. Chemotherapy with an oral fluoropyrimidine derivative is administered after surgery.^[4]^ The dismal prognosis is due, in part, to PDAC's chemoresistance and the inability of chemotherapeutic drugs to penetrate the dense fibrotic milieu associated with this cancer. Early detection may enhance the prognosis of the disease. However, only 10 %–15 % of patients are diagnosed when surgical resection is an option. Over 90 % of individuals had advanced PDAC.^[5]^ The efficiency of the nanomaterial depends on targeted transport of nanoparticles to tumour locations, tumour microenvironment and metastasis prevention, nanobiological interactions, host‐tumour immunological crosstalk, Enhanced Permeation and Retention (EPR) effects, and tumour heterogeneity. Nanomaterial production is also critical for the diagnosis and treatment of PC. These nanomaterials can be further functionalized to carry treatment enabling moieties. Various innovative techniques with functionalized nanoparticles including endogenous and exogenous stimuli‐responsive, surface conjugation, and encapsulation of nano‐drug systems have been effective in theranostics of pancreatic cancer.^[6]^ Thus, the potential of nanomedical techniques for early detection, efficacious delivery, and effective treatment is emerging as a viable option for pancreatic cancer diagnostics and treatment.

The reduced intratumoral vascular density from the extensive fibrotic characteristic of pancreatic cancer leads to dysfunctional vessels, which in turn causes hypoxia. Inadequate lymphatic and venous drainage also contributes to an increase in interstitial fluid pressure within pancreatic tumors. It has been shown that, pancreatic cancer stroma not only impedes the penetration and delivery of chemotherapeutic drugs but also accelerates their metabolic inactivation, resulting in an exceptionally poor response to treatment. Tumor development, chemoresistance, invasion, and metastasis are further enhanced by the reciprocal interaction between PSCs and the tumor cells. On the other hand, the stroma may also be the potential target in precision diagnostic imaging and therapeutic drug therapy aimed at disrupting the milieu that fosters tumor growth and metastasis.^[7]^ In a recent study, passivated nanowire biosensors were designed to enable the direct and rapid detection of the biomarkers for ovarian cancer (insulin‐like growth factor‐II and cancer antigen‐125) from human whole blood. The detection limits were significantly lower than the levels clinically relevant for diagnosis.^[8]^ High sensitivity and selectivity label‐free glycan analysis from pancreatic cancer cell lysates was made possible by the NanoMonitor, a tool that integrated nanoporous alumina membranes onto microfabricated silicon platforms.^[9]^ The enhanced multiplex, sensitivity, and specificity of nano‐diagnostics for cancer biomarkers can monitor treatment responses and early recurrences, undetectable by traditional assays.

The administration of an extraneous imaging agent improves the detection limit of MRI or CT imaging by enhancing the imaging signals. A contrast imaging agent is used in more than one‐third of MRI scans. Limited specificity and quick renal elimination were two drawbacks of the first generation of organic contrast agents. Contrast compounds in a nanoparticle formulation have been developed to overcome the above limitations. Incorporating contrast agents into nanoparticles can lead to easier functionalization through moieties targeting, increased sensitivity over tiny organic contrast agents, and enhanced biodistribution.^[10]^ Hybrid systems and multifunctional nanoparticles have also demonstrated significant promise. These nanomaterials have the potential to be therapeutic agents and exhibit increased signal amplification, which enhances diagnostic and imaging sensitivity. A study has described the imaging and treatment of colorectal cancers in mice using a hybrid nanoparticle of gold and iron oxide.^[11]^

Numerous chemotherapy drugs have adverse pharmacokinetics, low tumor absorption, and crippling off‐target toxicity. The effective use of nanotechnology as chemotherapeutic drug carriers is a strategy to address these issues. The comprehensive design of nanoparticles for the delivery of chemotherapeutic drugs has improved the drug's solubilization and lengthened its half‐life and stability in circulation. Additionally, multi‐drug resistance efflux pumps present on the surface of most tumor cells can be avoided by nanoparticles encapsulated with chemotherapeutic drugs.[^12^, ^13^] Nevertheless, owing to the simplicity to functionalize the surfaces of nanoparticles with targeting moieties (antibodies, aptamers, and small molecules) for improved binding affinity and specificity toward the tumor, nanotechnology has the potential to advance molecular‐targeted imaging, diagnosis and treatment in pancreatic cancer, despite possible drawbacks with passive tumor targeting.

## 2 Current Diagnosis and Therapies

Pancreatic cancers are hypovascular in nature and usually visualized with contrast imaging. The ideal investigation is a pancreas contrast computerised tomography (CT) scan with arterial and venous phase enhancement, which can assess local and regional disease extent. Thin slice cuts and high quality photos enable improved observation of vital vasculature, which determines tumour resectability.^[14]^ However, it may be difficult to distinguish pancreatic tumours from normal surrounding pancreas or chronic pancreatitis due to delayed amplification of pancreatic tumours. Contrast CT is also inadequate for detecting liver metastases and early lymph node metastases. Magnetic Resonance Imaging (MRI) and Endoscopic ultrasonography is critical in pancreatic cancer detection and management. Positron emission tomography (PET)/CT scans have also been helpful in detecting metastatic disease prior to surgical resection. It can be utilised to determine the tumour's depth. It can also be used to guide tiny needle aspiration biopsy to acquire tissue diagnosis without the need for invasive surgery. Endoscopic retrograde cholangiopancreatography (ERCP) is vital for visualisation of the biliary tree, allowing the implantation of stents to relieve biliary blockage in the majority of pancreatic malignancies.^[15]^

Surgery is the gold standard therapy for patients with resectable PC. Depending on the location of the tumour, the operational treatments may include cephalic pancreatoduodenectomy, distal pancreatectomy, or total pancreatectomy. Even after complete resection of the tumour, the prognosis for patients with early pancreatic cancer is dismal. Postoperative combination chemotherapy of fluorouracil with leucovorin or gemcitabine, which is routinely used to treat advanced pancreatic cancer, has been proven in studies to improve progression‐free and overall survival. Another clinical trial found that combining gemcitabine with fluorouracil as a continuous infusion and radiation therapy led in a trend toward enhanced overall survival among patients with pancreatic head tumours, however the increase was not statistically significant.^[16]^

In patients with localized disease, surgical excision is the only potentially curative therapy. Local and systemic recurrences are prevalent following a pancreaticoduodenectomy or total pancreatectomy. This failure pattern proves that both systemic and local adjuvant therapy may improve survival. Neo‐adjuvant therapy aims at improving of local control and survival by tumour volume reduction and achieving complete resection.^[17]^ For non‐resectable advanced and metastatic pancreatic cancer, palliative therapy and supportive care are the only options available to manage the disease. Adjuvant and Neo‐adjuvant therapies are also done to relieve the pain, improve survival and prevent progression of the disease. Most of the pancreatic cancers are diagnosed in the advanced stage that limits cure.^[18]^

## 3 Pancreatic Cancer Molecular Targets and Biomarkers

### 3.1 Genetic Mutations

The onset and progression of pancreatic cancer is influenced by both hereditary and epigenetic factors. According many sequencing studies, the four primary drivers of pancreatic cancer, KRAS, CDKN2 A, TP53, and SMAD4, have been implicated in its initiation. Involvement of twelve genetically altered core signaling pathways were discovered through whole exome sequencing, including those that control apoptosis, DNA damage, the G1/S phase transition, hedgehog signalling, homophilic cell adhesion, integrin signaling, c‐Jun N‐terminal kinase signaling, KRAS signaling, invasion control, small GTPase‐dependent signaling, and Wnt/Notch signaling. The main characteristics of pancreatic carcinogenesis can be determined by deregulation of these fundamental mechanisms.^[19]^

#### 3.1.1 KRAS Pathway Mutation

KRAS is a transmembrane GTP‐binding protein that regulates a number of biological processes, including cell motility, proliferation, and survival. Variations in KRAS's active and inactive states result in alterations to the intracellular signalling pathway. Nucleotide exchange factors (GEFs) that bind to GTP activate KRASs, whereas GTPase‐activating proteins (GAPs) that bind to GDP inactivate it. KRAS initiates the RAF/MEK/ERK and phosphoinositide 3‐kinase/protein kinase B (PI3 K/AKT) signaling pathways in its active state.^[20]^

##### 3.1.1.1 KRAS

An early oncogenic event in pancreatic cancer is KRAS mutation. In 25 % of pancreatic intraepithelial neoplasia (PanIN)‐1 A and 38 % PanIN‐1B, the KRAS mutation is easily identifiable. These results suggest that KRAS mutation likely represents an early and initiating event in human pancreatic cancer.^[21]^ The KRAS gene was mutated in 92 % of the cases in a cohort analysis of 109 pancreatic ductal carcinomas that underwent whole‐exome sequencing. It was also found that codon 12 was involved in majority of KRAS mutations.^[22]^ Many gene knockdown studies in multiple cancer cell lines utilising RNAi and CRISPR techniques have demonstrated that KRAS mutant pancreatic adenocarcinoma cells are more sensitive to the loss of the KRAS oncogene than KRAS WT cancer cell lines. These results show progression of pancreatic carcinoma is highly dependent on the KRAS oncogene mutation. Because KRAS mutant cells activate negative feedback loops that down‐regulate receptor signaling, it is possible that growth factor signaling is unable to reverse the stable KRAS dependency in pancreatic carcinogenesis.^[23]^ In a study, *Ela^5^‐Kras* ^*G12D*^ transgenic mice that target the pancreatic acinar cells were created. These mutant transgenic mice had ductal shaped preinvasive pancreatic neoplastic lesions, indicating the implication of Kras mutations in the onset of pancreatic ductal carcinoma.^[24]^ Another study involved mating of *LSL‐Kras* ^*G12D*^ allele‐carrying mice with either transgenic *Ptf1a‐Cre* mice or *Pdx1‐Cre* mice. The Pdx1 and Ptf1a are significant transcription factors involved in the development of pancreas. *PDX1‐Cre; LSL‐Kras* ^*G12D*^ mutant mice produced ductal lesions resembling human invasive pancreatic cancer. These lesions developed into invasive and metastatic PDAC within a year at low frequency. The cancerous tumour in the mouse model was strikingly similar to invasive PDAC in humans.[^24^, ^25^]

Acute KRAS silencing in KRAS‐mutant pancreatic cancer cells by RNAi or CRISPR caused cell cycle arrest, apoptosis, and reduction of anchorage‐independent proliferation in vitro, as well as reduced tumour growth and metastasis in xenograft models. Multiple Ras effector pathways, including the MAPK and PI3 K pathways, were downregulated when mutant KRAS is silenced. Therefore, inhibitors that target these Ras effector pathways could show selectivity for KRAS mutant pancreatic cancer cells.^[26]^ In another study, the MEK and ERK inhibitors, however, did not exhibit the same level of selectivity in KRAS mutant cells in large‐scale drug sensitivity experiments.^[27]^

##### 3.1.1.2 PTEN

PTEN (phosphatase and tensin homolog) is a crucial tumour suppressor and a tyrosine phosphatase type I member. PTEN is recruited from the cytosol to the membrane during the active phase after being dephosphorylated at its C‐terminal region, preventing PIP2 from hydrolyzing into PIP3. PTEN therefore blocks the PI3 K signalling pathway. Due to PIP3 buildup brought on by PTEN depletion, the PI3 K pathway becomes hyperactive. Genetic mutations, post‐translational changes, and epigenetic processes all cause PTEN to lose its functionality.^[28]^ In a study, Pten disruption exclusively in adult pancreatic ducts was created in mice. These mice produced intraductal papillary mucinous neoplasms (IPMN), and 31.5 % of those IPMNs were found to be invasive and metastatic. The pancreatobiliary subtype associated invasion was also linked to spontaneous Kras mutations. According to these findings, PDAC is initiated and develops mostly through the RAF/MEK/ERK and PI3 K/AKT signalling pathways.^[29]^

##### 3.1.1.3 B‐RAF

B‐RAF belongs to the RAF kinase family, which controls the MAPK signaling pathway. However only 3 % of PDAC cases had the B‐RAF^V600E^ mutation. Mutations in KRAS and B‐RAF are independent of each other.[^22^, ^30^, ^31^] In a study, BRAF^V600E^ was expressed in the adult pancreas of Pdx1‐CreERT2; Braf^CA/+^ mice under the control of a conditionally active Cre recombinase driven by the Pdx1 promoter. Near‐total replacement of the exocrine pancreas with PanIN lesions was observed due to the pancreatic expression of BRAF^V600E^. The study also expressed PIK3CA^H1047R^ mutation from the endogenous PIK3CA locus following Cre‐mediated recombination in Pdx1‐CreER^T2^; Pik3ca^lat−H1047R^ mice. However, up to six months, these models lacked evident PanIN lesions or other pancreatic abnormalities. Therefore, according to these findings, PanINs can be initiated by mutationally activated BRAF^V600E^ but not by PIK3CA^H1047R^.^[32]^

##### 3.1.1.4 PIK3CA

*About 3–5 % of the patients with pancreatic cancer carry PIK3CA* mutations. The catalytic subunit p110α of PI3 K is encoded by PIK3CA. All solid tumours have the PIK3CA H1047R mutation more frequently than any other mutation. Numerous downstream targets are phosphorylated due to the constant activation of the PI3 K pathway and less than 1 % of PDACs have PIK3CA mutations. But, somatic PIK3CA mutations have frequently been observed in pancreatic intraductal tubular papillary neoplasm (ITPN), an uncommon subtype of premalignant pancreatic lesion that differs from intraductal papillary mucinous neoplasm (IPMN).[^33^, ^34^, ^35^] However in an earlier study, two mouse models were produced that expressed pancreatic PIK3CA that was constitutively active. They produced Pdx1‐Cre transgenic mice with Pik3ca^p110*^, Pik3ca^H1027R^, or Pik3ca^p110*^ mice, which each had a conditional allele expressing an active PIK fusion protein. PanINs were discovered in the first model at 10 days, and invasive pancreatic ductal adenocarcinoma appeared at 20 days. Because the PI3 K pathway was activated to a lesser extent in the second model, PanINs and invasive malignancy formed more slowly. These Pik3ca mutant pancreatic tumours were found to share morphological similarities with Kras mutant models, highlighting the significance of PI3 K signalling in the carcinogenic potential of pancreatic cancer. Additionally, these tumours exhibited activation of ERK1/2 signalling, which may be downstream of PI3 K signalling as it initiates early in the tumorigenic process.^[36]^

#### 3.1.2 MYC

MYC belongs to a vital transcription factor family, which controls metabolism, cell proliferation, and differentiation. In healthy cells, its expression is strictly regulated, but it is overexpressed in human malignancies. MYC amplification occurs frequently (17 %) in pancreatic acinar cell carcinomas, which are unique and aggressive tumours. Additionally, MYC expression was found to be elevated in a subset of PDAC.[^37^, ^38^] According to a study, Myc is a crucial mediator of the metabolic alterations caused by Kras mutation and is an important driving factor for the survival of pancreatic cancer cells.^[39]^ Another study revealed that, Myc interacts with PIN1 to promote NRF2 expression, which protects pancreatic cancer cells from KRAS‐induced mitochondrial respiratory damage.^[40]^

A study found that between 2 and 7 months of age, mixed acinar/ductal pancreatic adenocarcinomas was induced by Myc expression under the elastase (Ela) promoter. Another study created a new paradigm that permits the temporally and spatially regulated expression of Myc in pancreatic progenitors and derived lineages of exocrine cells. Additionally, it was shown that upregulating Myc on its own triggered the development of ductal precursor lesions and, after a brief latency period, ductal adenocarcinomas were formed. Furthermore, it was demonstrated that post ablation of Myc, certain cancer cells remained quiescent despite a macroscopically complete regression of the primary and metastatic tumours. Following this a re‐expression of exogenous Myc in the cells promptly caused recurrence of pancreatic cancer. This study emphasises the significance of advanced eradication techniques for remnant cancer cells.[^41^, ^42^] Another study created the KPCXY (Pdx1CreER; Kras^G12D^; Trp53^fl/+^; Rosa^confetti/YFP^) model, using multiplexed fluorescence‐based labelling to monitor the several primary tumour cells lineages during metastasis. They discovered that increased Myc expression promotes metastatic spread by employing tumor‐associated macrophages. Single cell analysis of a paired primary and metastatic tumour of PDAC patients revealed an enrichment of MYC‐amplified subclones in metastatic lesions compared to the primary cells.^[43]^

#### 3.1.3 GNAS

The GNAS gene family has numerous G protein members, including as GNAS, GNA11, and GNAQ, which encode the Gαs, Gα11, and Gαq subunits respectively. The GTPase activity of these genes is compromised by oncogenic mutations, resulting in constitutively GTP‐bound active forms and prolonged downstream signalling.[^44^, ^45^] IPMNs lesions eventually result in PDAC. Histologically, IPMNs are distinguished by dilated mucinous pancreatic ducts that are bordered by columnar mucin‐producing cells that exhibit papillae with fibrovascular cores. R201 C and R201H are the most typical GNAS mutations found in IPMNs. A study that examined 132 samples of IPMNs using a novel ligation test discovered that 66 % of the samples had GNAS mutations, 81 % had KRAS mutations, and 51 % had both GNAS and KRAS mutations.^[46]^ According to another study, 172 IPMNs had 48 % GNAS mutations, 56 % KRAS mutations, and 31 % had both GNAS and KRAS mutations.^[47]^ GNAS mutations were found in invasive tumours, high‐grade tumours, and low‐grade tumours. Contrarily, typical PDACs do not contain GNAS mutations.[^46^, ^48^] The pathogenesis of IPMN is most likely initiated by the GNAS mutation rather than progression due to it.

#### 3.1.4 LKB1

LKB1/STK11 (liver kinase B1/serine‐threonine kinase 11) encodes an important serine‐threonine kinase and controls downstream kinases that are involved in the regulation of cellular response to energy stress and the development of cell polarity.^[49]^ LKB1 was first discovered as a tumour suppressor gene linked to Peutz‐Jegher Syndrome (PJS).^[50]^ PJS patients had a 132‐fold higher chance of acquiring pancreatic cancer than the general population.^[51]^ About 4–25 % of IPMN harbour the deleterious mutation of LKB1. Some studies reveal that IPMN in the pancreatic ducts could originate from the synergism of Kras^G12D^ and LKB1 mutations.^[52]^ In one study, conditionally expressed Kras and Lkb1 mutations in adult pancreatic ducts were used to create a mouse model. The researchers established that activating Kras^G12D^ mutation and Lkb1 inactivation led to IPMN, primarily of the gastric type, and shared some characteristics with human IPMN.^[53]^ These findings imply that IPMN initiated due to mutant KRAS is controlled by oncogenic GNAS activation and LKB1 inactivation.

#### 3.1.5 SMAD4

The SMAD4 belongs to the SMAD family of transcription factor, which mediates the signal transduction of TGF‐β, bone morphogenetic protein (BMP), and activin. About 31 % of PDAC have SMAD4 inactivating mutations, which are typically linked to high‐grade PanIN lesions. Genetic inactivation of SMAD4 may happen late in the development of mucinous cystic neoplasms (MCN), a precursor lesion of PDAC. Most benign MCNs do not exhibit SMAD4 mutations; however protein expression is frequently reduced in invasive malignancies derived from MCNs. In a study, mice carrying either the Pdx1‐Cre or Ptf1a‐Cre gene were crossed with mice carrying the conditional knockout allele of Smad4 (Smad4^lox^). PanINs developed more rapidly and tumours replicating human gastric type IPMN occurred relatively fast when Kras^G12D^ and Smad4 deficiency were present together. Smad4 loss was also found to promote PDAC development in KIC mice.

### 3.2 MicroRNA Markers

PC tissues exhibit distinct miRNA profiles that differ from normal tissues, chronic pancreatitis tissues, and other digestive cancer tissues. Furthermore, miRNAs and other molecular abnormalities can accumulate in the duct cells of invasive PC. miRNAs isolated from pancreatic tissue, pancreatic juices, blood, plasma, sera, bile, saliva, and faeces of PC patients have demonstrated potential diagnostic usefulness. Circulating miRNAs distinguished the others and could account for the most accurate diagnostic biomarkers.[^54^, ^55^, ^56^]

A study found that miR‐1290 demonstrated useful diagnostic performance and outperformed CA19‐9 in identifying patients with low‐stage PC from controls.^[57]^ To show the diagnostic use of plasma miRNAs, another study conducted a multicentre trial. The findings demonstrated that miR‐486‐5p has diagnostic relevance in distinguishing PC patients from healthy individuals or those with chronic pancreatitis.^[58]^ Although the potential diagnostic use of circulating miRNAs is encouraging, their limitations must be taken into consideration. First, there isn't a well‐established internal control for RTPCR analysis of circulating miRNA levels. Second, the total amount of miRNA in serum and plasma is minimal compared to that isolated from tissues or cell lines, and the current detection method is labour‐intensive and inaccurate. It is necessary to create new detection techniques that can efficiently analyze miRNA, improve detection's sensitivity and specificity, and require fewer amplification stages before detection. However, the source and mode of extracellular cancer‐associated RNA are yet unknown.^[59]^

### 3.3 Protein Biomarkers

#### 3.3.1 Mesothelin

Mesothelin is a membrane protein usually expressed in pleura, peritoneum, and pericardium mesothelial cells. Mesothelin levels were found elevated in a variety of malignancies, including mesothelioma, non‐small cell lung cancer, ovarian cancer, and pancreatic cancer. Mesothelin plays an important role in cell survival, migration, invasion, and tumour growth in previous studies.^[60]^

Silencing the expression of mesothelin inhibited growth of pancreatic tumour cells, suggesting it could be used as a potential treatment of PDAC. Mesothelin is a new marker for pancreatic adenocarcinoma identified by gene expression analysis. Mesothelin overexpression has potential diagnostic, imaging, and therapeutic implications. The mRNA expression of Mesothelin was confirmed by in situ hybridization and RT‐PCR in resected PDAC and pancreatic cancer cell lines respectively.^[61]^

#### 3.3.2 Urokinase Plasminogen Activator and Receptor

Urokinase plasminogen activator (uPA) belongs to the serine proteinase family that converts plasminogen to plasmin which then enables its protease activity. Its function involves tumour growth, invasion metastasis, and angiogenesis by activating matrix metalloproteinases and degrading of extracellular matrix. Increased levels uPA indicates poor prognosis in many cancers especially PDAC.^[62]^

Urokinase Plasminogen Activator Receptor (uPAR) belongs to a Ly6/uPAR/neurotoxin protein domain family which is a heavy glycosylated transmembrane protein with three homologous domains (DI, DII, and DIII). It has a three‐finger Ly6/ uPAR (LU) domain structure with characteristic disulphide bridge pattern. The external domain has a cavity available for Urokinase Plasminogen Activator (uPA) ligand binding.^[63]^ Soluble uPAR (suPAR) is upregulated and expressed in urine of patients having ductal pancreatic cancer with poor prognosis.^[60]^

#### 3.3.3 Insulin Growth Factor‐1 Receptor

A large percentage of cancer cells have an increased level of the Insulin‐like Growth Factor‐1 Receptor (IGF‐1R). In pancreatic cancer cell lines (PANC‐1 and HPAC) and human pancreatic adenocarcinoma tissues, the IGF‐1R was discovered to be abnormally over expressed. Effective IGF‐1R silencing was found to reduce proliferation and anchorage independent growth, suggesting a potential for IGF‐1R targeted therapy to prolong the lives of PDAC patients. Recent research has demonstrated that IGF‐1R is a crucial and significant driver of EMT‐related events in a small number of malignancies. Because of the greater possibility of metastasis, it was discovered that overexpression of IGF1R is actually linked to higher mortality in the majority of cancer patients. The motility and migratory ability of PDAC cells were also decreased when IGF‐1R was inhibited. As a result, when IGF‐1R is silenced, PDAC's invasive properties are greatly reduced. Soft agar experiments demonstrated that inhibiting IGF‐1R prevents pancreatic cancer cells from forming colonies in vitro. IGF‐1R‐silenced cells were shown to induce apoptosis, as shown by Western blot and flow cytometric analyses. Together, these effects have a strong inhibitory effect on pancreatic cancer cell proliferation and metastasis.^[64]^

#### 3.3.4 Epidermal Growth Factor Receptor

Epidermal growth factor receptor (EGFR) is mostly over‐expressed in pancreatic cancer progression. Epidermal Growth Factor Receptor (EGFR) is a transmembrane glycoprotein belonging to the tyrosine kinase family. In a typical cell, EGFR is critical in initiating major signalling pathways. Additionally linked to other cancer‐related signalling pathways, EGFR also contributes to angiogenesis, apoptosis, and resistance to chemotherapy and radiation. EGFR is overexpressed in 30–89 % of cases of pancreatic ductal carcinoma.^[65]^ Targeted therapy with nanoparticle conjugated with epidermal growth factor (EGF) ligand depicted increased cellular uptake mechanisms and apoptosis of targeted cells. This improved efficacy of anti‐cancer treatment. Numerous studies have linked the presence of overexpressed EGF receptors and a poor prognosis in individuals with pancreatic cancer. According to a study, 30.4 % of people with PDA overexpress the EGFR, and these patients have lower survival rates and lymph node metastases.^[66]^ Another study demonstrated cytoplasmic overexpression of EGFR was found in 76 tissue samples from PDA patients, with the invasive component being more common (62 %) than the intraductal one. This study contrasted the invasive and intraductal components. These results suggested a relationship between the greater rate of pancreatic cancer evolution and the overexpression of EGF. This demonstrates that EGFR is a significant predicting factor.^[67]^

#### 3.3.5 Plectin‐1

Plectin is a 500 kDa protein is expressed in several cell types and tissues of mammals. Actin‐binding, central coiled‐coil and plakin repeat domains make up this protein, which can be spliced into 12 distinct isoforms. In cellular organisation and signal transduction, plectin performs a crucial multifunctional role and can be found in variety of intracellular locations depending on cell type. As a cytolinker, it binds and maintains membrane and cytoskeletal proteins. It has been demonstrated that plectin interacts with phosphatidylinositol‐4,5‐biphosphate (PIP2), integrin, and calmodulin. Plectin is also a scaffolding protein known to attach to the receptor for activated C kinase 1 (RACK1) and so modulate protein kinase C (PKC) signalling pathways.^[68]^ In a study, plectin was found to be a particular and prevalent cell surface target in pancreatic ductal carcinoma (PDAC) that stays cytoplasmic in healthy tissue using a phage‐based functional proteomic method.^[69]^ Another work using PDAC cell lines, flow cytometry, immunogold transmission electron microscopy, and plectin‐binding tests has convincingly shown cancer‐specific plectin (CSP) expression on malignant cells (Bx.Pc3, L3.6pl, Panc‐1), but normal pancreas cells (HPDE) are null for CSP.^[70]^ Plec1 differentiates malignant pancreatic disease from chronic pancreatitis. Even before invasion, Plectin‐1 (Plec1) is overexpressed in early pancreatic ductal adenocarcinoma (PDAC) and during pancreatic carcinogenesis. A study was conducted to analyse the over expression of Plec1 utilizing immunohistochemistry and Western blot analysis were used to assess samples of human PDAC, chronic pancreatitis, and healthy pancreas. Plec1‐targeting peptides (tPTP) were used as a contrast agent for single photon emission computed tomography in an orthotopic and hepatic metastatic mouse model of PDAC to confirm Plec1 as an imaging target. All PDACs were found to have positive Plec1 expression, although benign tissues had negative expression. Only 0–3.85 % of early PDAC precursor lesions (PanIN I/II) were found to misexpress it, but 60 % of PanIN III lesions overexpressed Plec1. Plec1 over‐expression was also observed in all metastatic foci assessed like peritoneum, liver, and lymph nodes. Plec1 is the first biomarker to distinguish between primary and metastatic PDAC by tPTP in‐vivo imaging and has the potential to identify preinvasive PanIN III lesions. After imaging, biodistribution investigations revealed that the primary liver metastases and pancreatic tumours maintained 1.9–2.9 times more tPTP than the normal liver and pancreas, respectively.^[71]^

## 4 Nanoparticles for Pancreatic Cancer Theranostics

**Figure 1:**
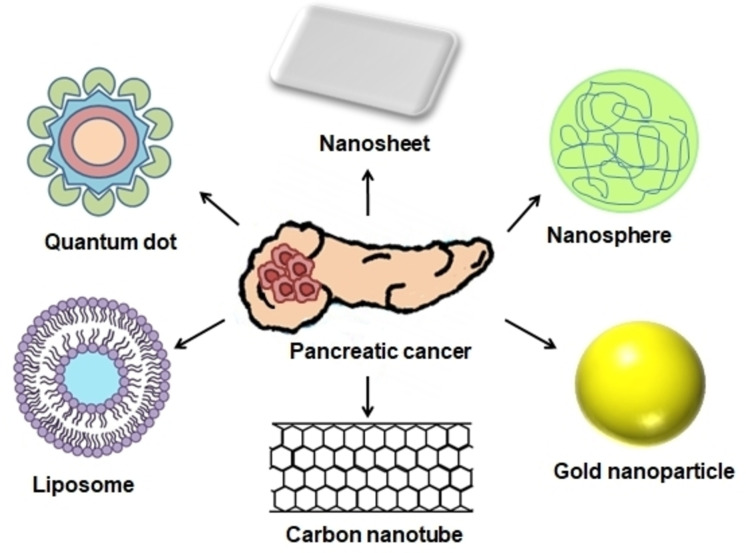
Nanoparticles used for pancreatic cancer theranostics.

Nanoparticles (NP) are important components in several nanobiomaterials systems for cancer detection. These particles are comprised of a range of materials, and each one has its own set of features that are designed to improve the detection of biomarkers. Nanoparticles have been approved for several clinical applications including diagnostics like imaging or biomarker testing (Figure 1). NPs are also used for therapeutic applications, or as a combination of diagnostic and therapeutic applications, known as therapeutic diagnostics.^[72]^

### 4.1 Nano‐Biomaterials Used in Pancreatic Cancer

According to the European Commission(EC), Nanomaterials are defined as “a natural, incidental or manufactured material containing particles, in an unbound state or as an aggregate or as an agglomerate and where, for 50 % or more of the particles in the number size distribution, one or more external dimensions is in the size range 1 nm–100 nm”.^[73]^ Nanomaterials operate at the nanoscale, and sometimes these particles range upto 500 nm.Nanomaterials can interact with biological systems to identify and monitor biological events more effectively and precisely during diagnosis and therapy. The characteristic of nanomaterials makes it coherent for theranostics of a variety of clinical conditions. One very important advantage is the nano‐size of these particles that enable them to circulate in the blood stream without obstructing any vessel and thus, prevents it from being cleared by the renal or complement systems.^[74]^

#### 4.1.1 Quantum Dots

**Figure 2:**
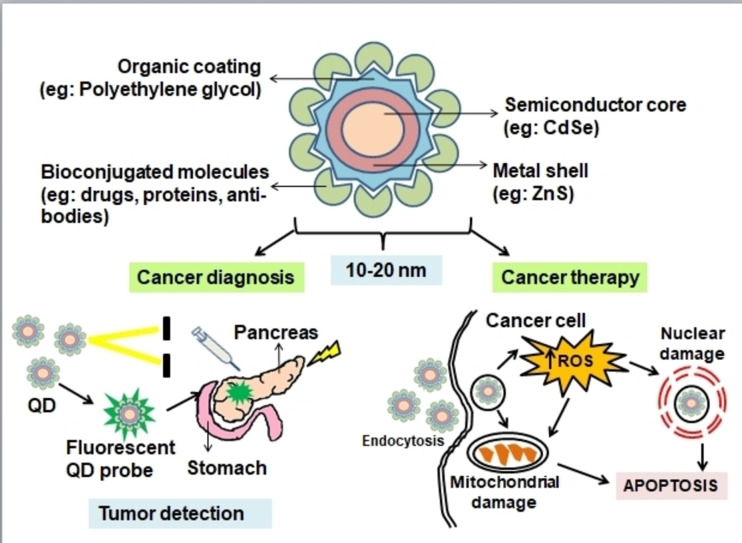
Quantum dots (QD) for pancreatic cancer diagnosis and therapy.

Quantum dots (QDs) are a type of nanoparticle that can be utilised in vitro and in vivo to detect cancer. These nanocrystals are made up of semiconductor particles with inorganic elements at their cores and metal shells around them. Quantum dots have a width of ten nanometres or less, and their strengths in cancer diagnosis and treatment including their application in cancer research is due to their unique properties.^[75]^ One of the important property of Quantum dots is the ability to produce unique fluorescence excitation wavelengths spanning from 400–2000 nm, due to their size and composition. QDs can be set to any colour to accommodate varied wavelengths, and hence they can be used to identify and track several biomarkers with just one light source (Figure 2). In addition, quantum dots have the advantage of becoming reusable and having a long lifespan, due to their resistance to fading. Quantum dots are also used to analyse microcirculation and inspect sentinel lymph nodes.^[76]^

#### 4.1.2 Carbon Nanotubes

Carbon nanotubes (CNTs) are hexagonal structures made up of self‐aligned single or double‐walled carbon molecules with diameters ranging from 0.3–100 nanometres. CNTs have several characteristics that make them ideal for usage in nanomaterials.^[77]^ CNT can work normally even when subjected to high temperatures or electrical currents because of its strong heat resistance and electrical conductivity. CNT also has a high tensile strength, which makes it resistant to permanent deformation caused by physical forces. Based on the different methods in identifying biomarkers, CNT may be separated into two categories: (a) depending on electrochemical signals generated by redox processes, (b) and the other is based on detection signals of field effect transistors formed by CNT surface charges.^[78]^

#### 4.1.3 Gold Nanoparticles

Gold NP is another type of metallic nanomaterial made of colloidal gold generally with drastic diagnostic potential. In aqueous solution, these nanoparticles mostly exist as gold nanospheres and have a bright ruby shade. The local surface plasmon resonance, in which the valence electrons of gold nanoparticles fluctuate with incident light at a certain frequency, is responsible for the optical features of gold nanoparticles. Some of the energy absorbed by gold nanoparticles is released as scattered light, which is used to image gold nanoparticles optically. The remaining energy decays in a non‐radioactive form, converting to heat that can be employed to kill cancer cells, making photothermal therapy possible. Gold nanoparticles have been discovered to be useful in a variety of tumour diagnosis and therapeutic applications.^[79]^

#### 4.1.4 Liposomes

Liposome is a type of nanomaterial made out of a lipid bilayer, which can be used to carry drugs to tumours. To enter tumour blood arteries and stay near to the tumour, liposomes rely on enhanced permeability and retention (EPR) effects. The structural properties of the circulatory system in solid tumours cause the EPR phenomenon. Nanoparticles make it easier to exude through the less tight endothelial connections. Nanoparticle retention rises in the tumour microenvironment due to insufficient lymphatic outflow. Liposomes are usually less than 200 nm in size to take advantage of the EPR effect and improve tumour retention. To improve cellular uptake in the target area, liposomes can be targeted with specific transport components.^[80]^

### 4.2 Nanomaterials Used for Early Diagnosis in Pancreatic Cancer

The protein alteration is the main hallmark of carcinogenesis and nanoparticle can concentrate on the human proteome alteration caused by cancer. These nanocarriers reach specific tumour cells and release drugs contained within. On coming in contact with organic body fluids like plasma etc., a coating of the biomolecule involved is formed as a shell covering the nanoparticle and this forms the biomolecular corona (BC). Mostly the biomolecule involved in cancer is protein and hence it is also known as protein corona (PC). As the protein alteration involved in PDAC varies with each patient, the PC formed if unique to each patient. The characterization of PC thus enables to detect changes in the protein concentration that otherwise cannot be detected by normal lab tests.^[81]^

The detection of circulating tumour cells (CTCs) is critical for tumour diagnosis. CTCs spread from the main tumour through the circulatory and lymphatic systems, forming secondary tumour colonies. CTC count has been utilised as a predictor of cancer development. However, CTCs are a very heterogeneous population, and identifying CTC subsets such as tumour stem cells with high metastatic potential is a difficult effort, but is critical for tumour diagnosis and management.^[82]^ CTCs with increased metastatic potential, such as CD24+ and CD133+ CTCs, have been discovered in live animals with the use of quantum dots, and may disclose detailed mechanisms of metastasis, such as tumour cell extravasation into blood arteries.^[83]^

Carbon nanotubes are used for the detection of specific biomarkers which is useful for the early diagnosis of cancer. One method of using CNT is by utilization of the electrochemical signals from the redox reactions. Both single‐walled and multi‐walled CNTs are used in a nanotube, where these nanotubes are lined in parallel and antibodies to specific biomarkers are attached to the surface. Another technique of detection is the field effect transistors method, in which nanotubes are linked to electrodes on both ends and functionalized to bind biomarkers. When these biomarkers bind to the nanotubes, a drop in conductivity is recorded, which is proportional to the amount of binding and quantification of biomarker is possible.^[78]^ PC genetic fingerprints have also been created using multi‐walled CNTs. Multiwalled CNT electrochemical sensing has been integrated with random amplified polymorphic DNA in studies. Variations in guanine and deoxyguanine triphosphate between DNA samples from PC patients and control patients were detected in peripheral blood.^[84]^

**Figure 3:**
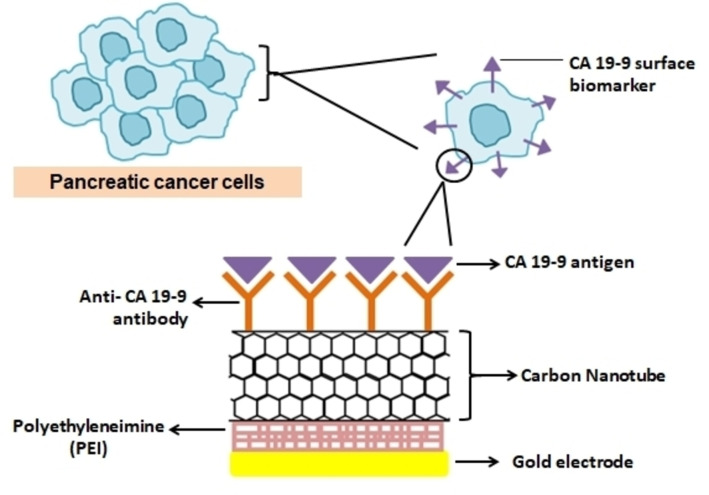
Carbon nanotube (CNT) biosensor for diagnosis of pancreatic cancer.

CA19‐9 is a tumour antigen is found in most of the pancreatic cancers. CA19‐9 was employed to create a new immunological sensor made up of carbon nanotubes, gold nanoparticles, and SiO2 nanoparticles.^[85]^ The nanosensor is made by coating the CNT's exterior surface with bovine serum albumin molecules. The gold nanoparticles are connected to the bovine serum albumin‐CNT, and the initial gold nanoparticles are nucleated by electrochemical deposition of gold. These preliminary processes provide a broad surface on which the CA19‐9 antibody can be mounted and used as a sensing element (Figure 3). SiO2 nanoparticles are modified as secondary antibodies for a sandwich immunoassay to increase signal and sensitivity of detection. A detection threshold 100 times lower than the ELISA standard currently used in clinical practise were revealed in experiments with various concentrations of CA19‐9 and hence suggest that this method could be more effective in diagnosing early PC.^[86]^

To enable improved detection of PC cells, ZnO QDs are also employed for the detection of CA19‐9 cancer biomarkers utilising an immunosandwich approach involving square wave extraction voltammetry and photoluminescence.^[87]^

### 4.3 Nanomaterials Used for Imaging in Pancreatic Cancer

Small molecules can be carried via methods for multimodality imaging using a single nano‐system. Normal healthy tissue imaging has the drawback of exhibiting autofluorescence, which interferes with signals from cancerous tissue. Quantum dots are made to combine with fluorescent characteristics in the near‐infrared wavelength, which eliminates autofluorescence interference. The danger of toxicity following injection is one potential issue with employing QDs *in vivo*. Although certain advances have been achieved to lessen the risk of toxicity, more research is needed to discover the best clinical treatments.^[75]^

Organically modified silica nanoparticles with a diameter of 20 nm were utilised to target PC cells. The fluorescent rhodamine B was used as the imaging agent, and the surface of these nanoparticles was functionalized with transferrin, stabiliser four, and antimycin. This method is simple to use and is appropriate for photobiological imaging studies.^[88]^ Mesoporous silica nanospheres were shown to be functionalized with the target ligand and covalently linked to the rare earth ion Gd (III) via a disulphide group in another investigation. In vitro, it was demonstrated to be an effective MRI contrast agent in PC models however, in vivo, the disulphide bond was swiftly digested.^[89]^

For tumour imaging, nontoxic, long‐acting fluorescent probes are required for non‐invasive gathering of tumorigenesis and metastatic information. To scan malignancies in vivo, a biocompatible near infrared ray (NIR) fluorescence probe containing glucose (Glc) on the surface of NIR Ag2Se quantum dots (NIR Ag2Se QDs) was used. For at least 7 days, these glucose‐functionalized Ag2Se QDs (Glc‐Ag2Se QDs) were seen *in vivo*. The probe was eliminated by the kidney, and the excretion capacity is favourable for *in vivo* imaging. Glc‐Ag2Se QDs were also used for targeted imaging of human breast cancer cells (MCF‐7) and imaging of SW1990 PC cells.^[90]^

### 4.4 Nanomaterials Used for Chemotherapy

Chemotherapeutics can limit tumour growth or prevent metastasis. Many cytotoxic drugs can be utilized as cancer chemotherapeutics but the major limitation is the lack of specificity of these drugs. The current strategy causes systemic toxicity and destroys the healthy surrounding cells. Chemotherapeutics also have issues with water solubility, non‐specific distribution, rapid removal from blood circulation, and drug resistance. Modification to drug delivery system is the only solution to these issues. Drugs are linked to the nanoparticle through covalent or non‐covalent bonds. Functionalized nanoparticle delivery techniques that are well‐designed can aid in drug delivery and overcome this problem.^[91]^

According to a recent study, a drug delivery nanosystem based on gold nanoparticles (PEGAuNPs) was able to deliver medications to tumour cells successfully. Anthracycline and tyrosine kinase inhibitors, Adriamycin and volitinib, were combined with gold nanoparticles and adjusted for size, stability, and shape. In human cells, the combination of PEGAuNPs, adriamycin, and varlitinib was beneficial, with PEGAuNPs inhibiting cancer cell proliferation while minimising damage to normal cells. On human leukemic cancer cells, Parvifloron D (PvD), a natural chemical isolated from the Plectranthus genus, displayed cytotoxicity and anti‐proliferation action. PvD is a water‐insoluble molecule, and employing albumin nanoparticles generated by desorption, was an effective technique for solving this problem. The cytotoxicity of the nanoencapsulated PvD was limited to PC cell lines. PvD was wrapped in nanoparticles that were moulded to a similar size (100–400 nm) and form after further optimization, which included glucose crosslinking. In the BxPC‐3 PC cell line, linking erlotinib to these PvD nanoparticles resulted in significant antiproliferative action.^[92]^ Overexpression of the extracellular matrix (ECM) in pancreatic ductal adenocarcinoma cells inhibits drug penetration into tumours and is linked to a poor prognosis. In a recent study, researchers found that collagenase, a proteolytic enzyme pretreated with a nanoparticle system dissolved the dense extracellular collagen matrix of PC and enhanced drug penetration in an orthotopic mouse PC model. This collagenase was enclosed by a 100‐nanometer liposome and this encapsulation prevented collagenase from premature inactivation thus extending its release rate at the target site, and therefore boosting the drug's efficacy. Degradation of the extracellular matrix, however, had no effect on the amount of circulating tumour cells or metastases.^[93]^

Gemcitabine (GEM) is a primary, discrete chemotherapeutic medication for PC, however after a few months, tumour cells become resistant to it. GEM has been shown to improve treatment outcomes in a number of clinical and preclinical trials, when used in conjunction with other chemotherapeutic agents. However, systemic toxicity is common with this form of combination therapy. As a result, new treatment regimens are needed to administer chemotherapeutic medication combinations to patients safely. Nanoparticles can be used to transport combinations of pharmaceuticals to tumour cells at the same time.^[94]^ Poor pharmacokinetics, thick fibrosis, and hypo‐vascularization limit gemcitabine administration to pancreatic ductal cancer. Activatable liposomes release drugs in response to local hyperthermia, improve serum stability and circulation while retaining the drug's capacity to diffuse within the tumour. The limited loading efficiency of liposomal gemcitabine was overcome by using copper (II) gluconate as a component to build a compound with gemcitabine at copper: gemcitabine ratios (1 : 4). Cell death and the development of apoptotic regions were observed in aggressive tumour model of murine pancreatic cancer.^[95]^

#### 4.4.1 Combination Chemotherapy

Nanoparticles can be loaded with two or more medications for simultaneous delivery, resulting in a synergistic therapeutic effect. Nanoparticles can be made to be hydrophobic, hydrophilic, or amphipathic, improving the solubility of hydrophobic drugs in blood plasma. A study found that chemotherapy photothermal combination therapy could boost chemotherapeutic medication efficacy in PC patients. The use of paclitaxel‐targeted polydopamine polymer microspheres in combination with programmable gold nanoparticles improved the efficacy of photothermal treatment in PC cell lines significantly.^[96]^

Drugs delivered by a nanoparticle has a longer blood circulation duration because it is not easily destroyed by enzymes or removed by the immune system.^[91]^ In the immune system, macrophages are responsible for particle clearance. Plasma opsonin proteins often cover nanoparticles, causing macrophages to identify and destroy the nanoparticle from the blood. To prevent macrophage phagocytosis, the nanoparticle can be functionalized with the biocompatible and non‐immunogenic hydrophilic polymer polyethylene glycol (PEG). This functionalization prevents opsonins from mobilising on the nanoparticle surface. The prolonged circulation period enhances medication distribution throughout the body.^[97]^

Due to the physical properties, nanoparticles can pass through membranes and epithelial layers. Another observed phenomenon is nanomedicine accumulation in benign and malignant tumours which is known as the enhanced permeability and retention (EPR) effect. This effect arises because most solid tumours contain blood arteries with faulty structure, resulting in increased vascular permeability and an adequate supply of nutrients and oxygen to the tumour for growth. If the nanoparticle is functionalized with a recognition molecule, such as an aptamer or an antibody, the cancer cell can endocytoze it. In drug delivery, the purpose of nanomedicine is to target the nanoparticle and deliver the chemotherapy into the cancer cell to reduce cytotoxicity in healthy cells.^[91]^

### 4.5 Targeted Gene Therapy and Sirna

Pancreatic cancer is often associated with the accumulation of mutations and genetic lesions, which leads to the activation of oncogenes and the inactivation of tumour suppressor genes. There is no specific therapy that targets mutant KRAS. Recently, mRNA targeting by RNA interference (RNAi) has been shown to be a highly effective alternative to the more widespread protein inhibition technique.^[98]^

**Figure 4:**
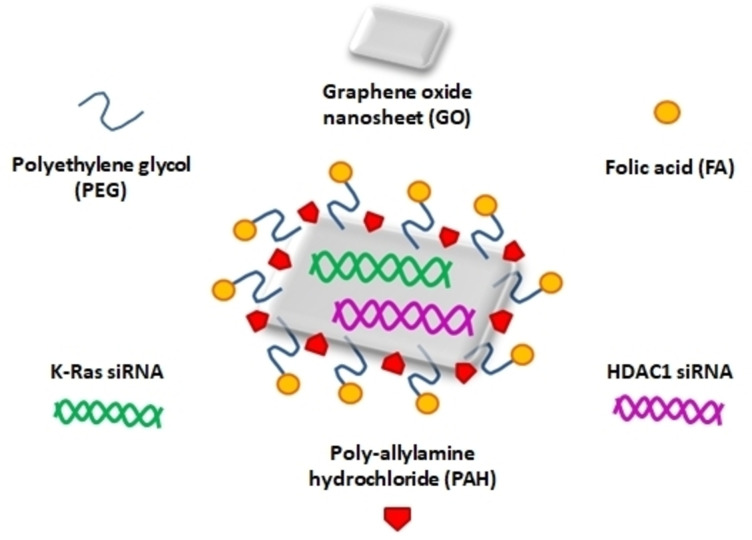
Functionalized Graphene oxide (GO) nanosheet for targeted gene therapy.

Mutated KRAS has been considered as a hallmark of pancreatic cancer since this protein is mutated in more than 90 % of PDACs. KRAS and HDAC1 gene mutations may be early events in the molecular pathogenic cascade that leads to PDAC and thus they are potential targets for pancreatic cancer gene therapy.[^99^, ^100^] A study used multi‐functionalized monolayer graphene oxide (GO) as a gene delivery vehicle to effectively co‐deliver HDAC1 and K‐Ras siRNAs (small interfering RNAs) targeting the HDAC1 gene and the mutant K‐Ras gene in pancreatic cancer cells MIA PaCa‐2. GO is a monolayer of graphite oxide that has hydrophilic surface groups like hydroxyl and carboxyl groups. Folic acid (FA), NH2‐mPEG‐NH2 (5 k), and Poly‐allylamine hydrochloride (PAH) were conjugated onto GO nanosheets to create functionalized GO nanoparticles (Figure 4).

**Figure 5:**
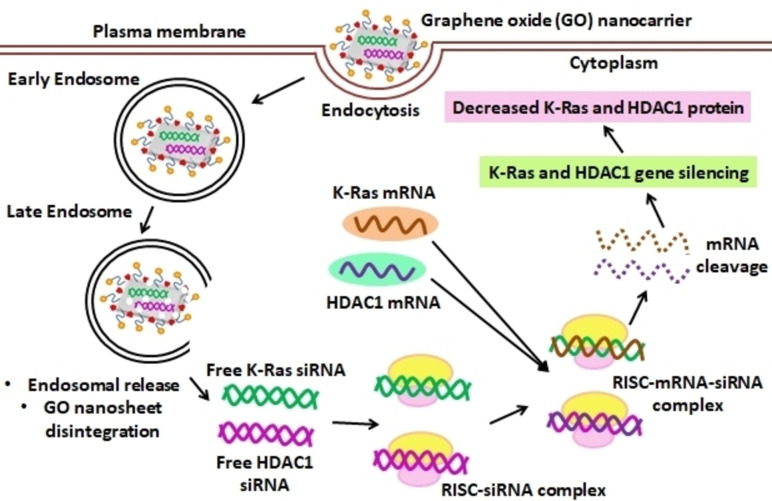
Mechanism of action of GO based nanocarrier for siRNA targeted therapy.

Biocompatible polyethylene glycol (PEG) polymers can functionalize these surface groups thus improving the loading capacity. The majority of pancreatic cancer cells are folate Receptor‐expressing (FR+) cancer cells and thus have the ability to uptake conjugates of folic acid with GO through receptor‐mediated. Therefore targeted medication delivery is possible and significant rate of internalization was observed in co‐delivery of HDAC1 and K‐Ras siRNA via PAH/FA/PEGylated GO nanosheets. In treated MIA PaCa‐2 cells, inactivation of HDAC1 and the K‐Ras genes led to apoptosis, inhibition of proliferation, and cell cycle arrest due to the dual gene silencing effects (Figure 5). The growth of in vivo tumour volume was significantly inhibited in a mice model, due to the synergistic combination of gene silencing and NIR light thermotherapy. Functionalized graphene oxide has therefore been developed into the best gene delivery methods.^[101]^

### 4.6 Photodynamic Therapy (PDT)

**Figure 6:**
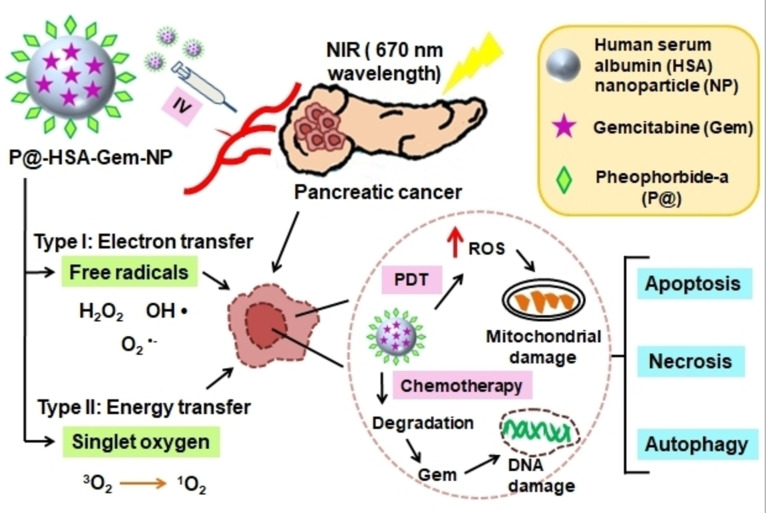
Triple functionalized nanoparticle for photodynamic therapy for pancreatic cancer.

The standard chemotherapy for pancreatic cancer is using gemcitabine (Gem) but its clinical advantages are jeopardised due to the short plasma half‐life and relative low concentration surrounding tumour sites.^[94]^ Albumin is an efficient drug carrier because it is biocompatible, biodegradable, and nontoxic. Pheophorbide‐a (P@) is a second‐generation PDT agent with a high singlet oxygen quantum yield.^[102]^ In PDT, photosensitizer (PS) is administered before being exposed to near‐infrared (NIR) radiation to cause cell death. The whole reaction produces harmful reactive oxygen species that can destroy cancer cells, including singlet oxygen and free radicals (Figure 6). A study created P@‐Gem‐HSA‐NPs, which supports PDT, controlled chemotherapy, and imaging, by encapsulating Gem inside P@‐conjugated human serum albumin (HSA). Through the enhanced permeability and retention (EPR) effect, these intravenously (IV) injected triple‐functionalized NPs could easily accumulate within the tumour site and metastatic lymph nodes. The PS P@ can produce optical fluorescence under NIR irritation, which is employed for early diagnosis and tracking drug administration. The liberated Gem and P@ will have a combination PDT and chemotherapeutic impact.^[103]^

## 5 Immunomodulation by Functionalized Nanoparticle

Extracellular matrix (ECM), tumor‐infiltrating lymphocytes, cancer‐associated fibroblasts (CAF), myeloid suppressor cells, regulatory T cells, and tumor‐associated macrophages (TAM) are all components of the unique tumor microenvironment (TME). Additionally, the TME is loaded with soluble proteins such transforming growth factor beta (TGF−), cyclooxygenase 2 (COX‐2), and epidermal growth factor (EGF). The interaction of these components results in an immunosuppressed TME, which supports the growth, development, and metastasis of the tumor while impairing the activity of T cells and Antigen presenting cells (APC).[^104^, ^105^, ^106^]

According to a study, fluorescent mesoporous nanoparticles (FMSN) were efficiently absorbed by human cancer cells and this cellular uptake necessitates energy expenditure and temperature‐dependent endocytosis. It has also been demonstrated that paclitaxel, an anticancer drug, was stored in FMSN and delivered to human cancer cells, which inhibited the growth of those cells. These findings highlight FMSN's value as a delivery system for anticancer medications. Currently, nanovalves attached to FMSN are being explored to allow for the regulated release of the anticancer medications that have been stored.^[107]^ Therefore, the use of functionalized nanoparticle will present a promising path for the creation of an efficient system for the delivery of cancer medication. The blood‐brain barrier (BBB), which prevents the majority of drugs from penetrating the brain, makes it difficult to treat brain metastases from most malignancies. Additionally, the BBB prevents nanomedicines from building up in the brain, necessitating the modification of nanomedicines to increase the effectiveness of brain targeting. Based on this condition, the researchers created terpolymer‐lipid hybrid nanoparticle (TPLN) in conjugation with cyclic internalizing peptide (iRGD), a surface‐modified nanoparticle that can pass through the blood‐brain barrier in mice with triple‐negative breast cancer brain metastases. This was followed by active targeting of tumor associated macrophages (TAM) in brain metastatic microlesions. Additionally, silica‐coated iron oxide nanoparticles (NF‐SIONs) can increase macrophage endocytosis absorption and cross the blood‐brain barrier with glioblastoma (GBM).[^108^, ^109^] It also opens new perspective of the possibility of using such modified nanoparticles to load pertinent immune activators to induce corresponding tumor immune activation, even though this study only showed that nanoparticles that can penetrate the blood‐brain barrier can target TAMs and thereby play a role in imaging tumors. Together, surface modification of nanoparticles allows it to cross the blood‐brain barrier and target immune cells in the brain.

Immune responses to tumors can be both non‐specific and specific to the tumor. The tumor‐specific immune response is triggered by the tumor antigens being taken up by APCs and converted into MHC‐antigen peptide complexes, which then activate downstream T cells. However, even though the majority of human cancers contain tumor‐associated antigens, these antigens lack the strength and specificity needed to trigger an efficient immune response. Fortunately, the body also has a second defense mechanism, the tumor non‐specific immune response, which is primarily made up of macrophages and natural killer (NK) cells.^[110]^

Endocytosis plays an important role in the uptake of functionalized nanoparticles by dendrtitic cells (DC). In the event that the internalized antigen is not released from the endosome into the cytosol, it will be delivered to CD4+ T cells via MHC‐II molecules for recognition and to trigger an immunological response involving CD4+ T cells. Therefore, it is essential to facilitate the release of endogenous antigens, which are presented to CD8+ T cells in the form of antigenic peptide‐MHC class I molecule complexes, from endosomes into the cytoplasm to cause immune cells induced tumor apoptosis. Enhancing the ability of antigen‐loaded nanoparticles to target DCs can result in DC stimulation.[^111^, ^112^, ^113^]

Nanoparticles have the ability to both directly activate T cells and indirectly promote T cell activation by increasing DC stimulation. One method to promote T cell activation is a nanovaccine made for tumor‐specific CD8+ T cell activation. Notably, tumor nanovaccines can be made using nanoparticle‐based artificial APCs (aAPCs), which imitate the antigen‐presenting and T‐cell activating capacities of natural APCs. A study created the surface functionalization of azide‐engineered leukocyte membrane‐coated magnetic nanoclusters with MHC−I peptide and anti‐CD28 antibody. Cytotoxic T cells that are specific for an antigen are expanded and activated by the modified nano‐aAPCs.^[114]^ Additionally, the surface coating of nanoparticles to create aAPCs can be done using the cell membrane isolated from DCs, facilitating direct cross‐priming of T cells.^[110]^

In recent years, creating immunostimulatory nanoparticles has become a hot topic in research because these particles can directly activate T cells, macrophages, and NK cells in addition to being efficiently internalized by APCs, to deliver cancer antigens and adjuvants and start an immune response that is specific to the antigens they contain. The necessary immunostimulatory effect can be achieved by combining serum proteins with the corona formation on the surface of nanoparticles. Additionally, surface‐modified nanoparticles can shield delivery molecules from off‐target impacts and loss of biological function during the process. Thus functionalized nanoparticles can stimulate immune cells such dendritic cells (DC), T‐ cells, natural killer (NK) cells, and macrophages.[^115^, ^116^]

## 6 Animal Model Studies

Peptide functionalized NP enables peptide mediated in vivo tumour targeting by NPs. This improves the interaction between NP and tumour interaction. A study targeted the pancreatic tumour cells using a squalene based nanoparticle conjugated with a new ligand CKAAKN peptide.^[117]^ CKAAN peptide has been proven to be an efficient homing device within the microenvironment of pancreatic diseases. This was identified using phage display in mouse models with pancreatic ductal tumorigenesis.^[118]^

In this study, RIF‐Tag 2 transgenic mouse models bearing pancreatic tumour was used. A squalene based NP conjugated with gemcitabine (SQdFdC) and a squalene based NP conjugated with gemcitabine and CKAAN (SQdFdC/SQCKAAN) peptide was injected independently in the mouse model via intravenous route. It was observed that the peptide functionalized NP had unique selectivity and multiple mechanisms of action. The peptide functionalized NP improved therapeutic efficacy of gemcitabine in the mouse model due to active targeting and tumour regression was noticed. It also increased the apoptotic rate by 82 % compared to the control and independent gemcitabine administration and decreased the cancer proliferation. The FACS analysis enabled to confirm the strong affinity of peptide functionalized NP to the tumour vasculature compared to the non‐functionalized NP. A decrease in the tumour volume and impaired tumour growth was also noticed. This SQdFdC/SQCKAAN NP also has the ability to act as Wnt‐2 mimetic and bind to the frizzled receptor(FZD)thus inhibiting the tumour progression as Wnt signalling is almost always overexpressed in pancreatic cancers.^[117]^

Targeted PEGylated mesoporous silica nanoparticle was used as a carrier to deliver curcumin and gemcitabine, as an adjuvant therapy. This was found to improve treatment outcome of pancreatic cancer patients. Mesoporous silica nanoparticles(MSN) loaded with curcumin(Cur) and conjugated with targeting moiety transferrin(Tf) which was coated with polyethylene glycol(PEG) was synthesized. The curcumin uptake was found to be 7 times higher with conjugated MSN compared to normal exposure. Conjugated MSN‐NH2‐Cur‐PEG−Tf exhibited 3 times higher cytotoxicity than free curcumin. The conjugated MSN‐NH2‐Cur‐PEG−Tf inhibited tumour growth and minimize distant metastasis to major organ sites when tested on MIA PaCa2‐ Human Pancreatic Cancer (PC) animal model. This increased sensitization effect of the pancreatic cancer cells both in‐vitro and in‐vivo which aided gemcitabine to kill higher percentage of the cancer cells by making the cells susceptible to DNA damage mediated cell death. The addition of gemcitabine as adjuvant drug along with the conjugated nanoparticle decreases disease burden for pancreatic cancer patients and increased cytotoxicity 1.4 times. The Enhanced Permeation and Retention (EPR) effect helps the NP to passively reach the tumour site. The ligand attached on the surface of NP targets the transferrin receptor overexpressed in pancreatic cancer cell as they require iron for growth and proliferation by supporting mitochondrial respiration.^[119]^

## 7 Clinical Trials

Paclitaxel was combined with human serum albumin in an aqueous solvent and pressed under high pressure to generate a 100–200 nm drug nanoparticle albumin bound paclitaxel (nab‐paclitaxel). On researching the effect of nab‐paclitaxel, one phase I/II study and one phase II study were discovered. One phase I/II study found that combining nab‐paclitaxel and gemcitabine chemotherapy was beneficial. Another research, which included patients with advanced pancreatic cancer, failed to demonstrate a convincing therapeutic impact of this medicine. In a phase I/II research, 67 patients were randomly assigned to one of three groups, with each group receiving various amounts of nab‐paclitaxel and gemcitabine on three days of every 28‐day cycle. They discovered that the dose‐limiting adverse effects were neutropenia and sepsis. The median overall survival was 12.2 months, and that the one‐year survival rate was 48 %. Patients’ metabolic activity was reduced by 79 % on average in all three treatment groups, with the group getting the highest dose of nab‐paclitaxel experiencing the greatest reduction.[^120^, ^121^]

A clinical experiment was done with the goal of delivering KRAS RNAi‐based medicines to the target site and achieving extended activity at a tolerated dose. A biodegradable nano‐polymeric matrix was used to encase anti‐KRASG12D siRNA (siG12D). siG12D was created to offer delayed and prolonged local medication release within the tumour over several months while protecting the siRNA drug from degradation. A typical endoscopic ultrasound (EUS) biopsy procedure can be used to insert and implant it into a pancreatic tumour. In combination with already available chemotherapeutics like Gemcitabine or Paclitaxel, siG12D was employed as a novel treatment approach targeting mutant KRAS. Serial histological examinations revealed that the medication often covered the whole tumour mass within one week. In the total tumour mass, direct effects of KRAS expression inhibition on mRNA and protein levels, as well as indirect effects of tumour growth inhibition and tumour cell death induction, were found. Tumour growth was reduced in various in‐vivo mouse models, and survival rates were dramatically enhanced following siG12D implantation. Apoptosis of tumour cells and tissue necrosis were reported to be extensive in siG12D‐treated tumour tissue after one week. SiG12D slows tumour cell migration and inhibits Epithelial to Mesenchymal Transition (EMT) in addition to having local effects on the tumour (Table 1).

**Table 1:** List of current and past clinical trials involving functionalized nanoparticle for pancreatic cancer therapy.

Functionalized nanoparticle	Description	Phase	Reference
Nanoparticle albumin (NEOLAP)	Neoadjuvant chemotherapy withCombination of nanoparticle albumin (nab) associated paclitaxel with gemcitabine and folfirinox infusion for locally advanced and non‐metastatic pancreatic ductal adenocarcinoma.	Phase 2	NCT02125136^[122]^
Albumin stabilized paclitaxel nanoparticle	Perioperative combination chemotherapy of Paclitaxel Albumin‐Stabilized Nanoparticle with Fluorouracil, Gemcitabine Hydrochloride, Irinotecan Hydrochloride and Oxaliplatin infusion for patients with resectable pancreatic adenocarcinoma.	Phase 2	NCT02562716^[123]^
Irinotecan liposome	Administration of liposomal Irinotecan, Fluorouracil and Leucovorin for patients with refractory advanced high grade neuroendocrine carcinoma of pancreatic origin to identify the efficacy of circulating tumor DNA as potential biomarker	Phase 2	NCT03736720^[124]^
Pathotropic Rexin G nanoparticle	Gene therapy by infusion of Rexin‐G in patients with locally advanced pancreatic cancer.	Phase 1/2	[125]
Liposome nanoparticle	Liposome conjugated with cisplatin and gemcitabine in patients with refractory and locally advanced pancreatic cancer	Phase 1/2	[126]
Paclitaxel conjugated micelle nanoparticle	NK105 micelle carrier system for paclitaxel was administered to patients with local and metastatic pancreatic cancer.	Phase 1	[127]

## 8 Conclusion

Pancreatic cancer is one of the lethal tumours that are often diagnosed in the advanced stage involving metastasis to surrounding organs and lymph nodes. Due to the molecular nature of pancreatic cancer, some surface proteins are overexpressed in cancer cells compared to healthy cells. It is conceivable to construct tailored and targeted therapeutics against these molecular markers by using nanoparticles functionalized with antibodies or aptamers. The current review highlights recent developments in functionalized nanomaterials for cancer theranostic applications for pancreatic cancer.

The key challenge is creating pre‐clinical models that can be converted into human pancreatic cancer models. The use of emerging techniques like CRISPR‐Cas9 for specific knock‐outs and various other multi‐plexed gene editing techniques may be helpful to overcome this challenge. Another major challenge would be preventing cytotoxicity due to the nanomaterials, which can be overcome by further dosage based studies and having a mechanism to eliminate the excess/unwanted nanoparticles based on their binding to tumor cells. Functionalized nanomaterials hold significant promise for pancreatic cancer theranostics, combining therapy and diagnostics into a single platform. However, there are several key challenges associated with their development and clinical application. The main challenge would be ensuring the nanomaterials are biocompatible and non‐toxic to avoid adverse reactions. Thorough preclinical testing to evaluate and mitigate toxicity and development of surface modifications to reduce immune response and improve biocompatibility. Penetration of the nanoparticle into the dense stromal environment could be another potential issue even though the nanomaterial has been functionalized. Employing strategies like tumor‐penetrating peptides or nanoparticles designed to respond to the tumor microenvironment like pH‐sensitive or enzyme‐responsive nanoparticles. The treatment can use nanoparticles to deliver conventional chemotherapeutics or new compounds like RNAi or suicide DNA genes. The development of photothermal and photodynamic therapies is another promising technology that involves the use of nanoparticles and has less negative effects than traditional therapies.

An ideal nanotheranostic system must be stability, have efficient payload capacity, nontoxic, biocompatible, easily modifiable, capable of endocytosis and specific efficient tumor targeting. This can aid in the effective and efficient evaluation of pancreatic cancer therapy with nanoparticles and chemo‐therapeutic combinations in early‐phase clinical trials, as well as the establishment of improved regulatory endpoints for pancreatic cancer nanomedicine.

## Author Contributions

Yoghalakshmi Nagarajan: Writing – conceptualization, review & editing, Writing – original draft, Natarajan Chandrasekaran: review & editing. Venkatachalam Deepa Parvathi: conceptualization writing – review & editing, supervision and finalization.YN and VDP the authors critically revised the work. The authors have read and approved the final version.

## Conflict of Interests

The authors have no relevant financial or non‐financial interests to disclose.

9

## Biographical Information

*Yoghalakshmi Nagarajan completed her B.Sc.(Hons). Biomedical Sciences at Sri Ramachandra Institute of Higher Education and Research, India. Her academic journey is driven by a strong desire to explore the field of cancer immunotherapy, where she aims to uncover innovative treatments that harness the body's immune system to fight cancer. With a clear vision for her future, she aspires to make significant strides in the field, ultimately improving research for better patient outcomes and advancing medical science. She is currently pursuing her Elite masters in Human Biology – Principles of Health and Disease, Ludwig Maximilian University, Munich*.

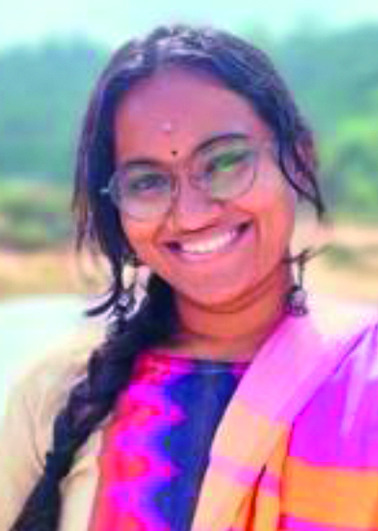

## Biographical Information

*With a passion to teaching and student affairs, Dr. V Deepa Parvathi has been an academician since 2006, contributing to innovative teaching methods for undergraduate and postgraduates across various disciplines of the university. She is the proud recipient of Young Scientist Award in 2019, Teaching Excellence Award 2020 and Shri PK Das Memorial Best Faculty Award (Senior Category – Biosciences) 2023. Her areas of research interests and expertise include Cancer Biology(gastric cancer and oral squamous cell carcinoma), Human Genetics(modeling neurodegenerative genetic disorders), Nutrigenomics(aging studies) and Nanogenotoxicology on animal models including Drosophila and Zebra Fish. She has to her credit several publications in journals of national and international repute. She has also authored five full length textbooks and three monographs published by renowned publishers. Her clinical focus to the diagnostic division of Department of Human Genetics included handling Prenatal diagnosis, Cancer cytogenetics and FISH cases from 2014–2019.Currently Associate Professor in the Department of Biomedical Sciences, Sri Ramachandra Institute of Higher Education and Research, India*.

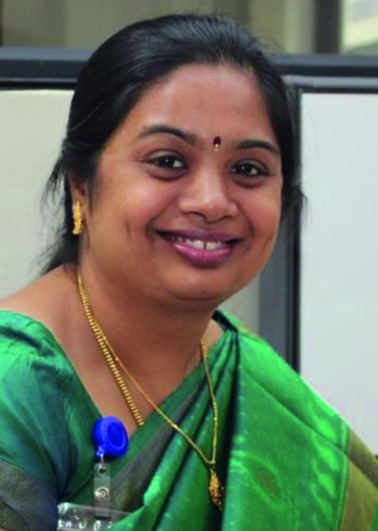

## Data Availability

Not applicable.
